# Current Treatment of Primary Biliary Cholangitis and Primary Sclerosing Cholangitis: A Comprehensive Review

**DOI:** 10.5152/tjg.2026.26267

**Published:** 2026-06-19

**Authors:** Victoria E. Abadi-Ron, Ana Marenco-Flores, Natalia Rojas-Amaris, Fernando Munguía, Javier Polo-Ibarra, Behnam Saberi, Vilas R. Patwardhan, Alan Bonder

**Affiliations:** 1 Division of Gastroenterology, Hepatology, and Nutrition, Beth Israel Deaconess Medical Center, Harvard Medical School, Boston, USA; 2 Department of Emergency Medicine, Boston Medical Center, Boston University, Boston, USA

**Keywords:** Cholestatic liver disease, current treatments, liver transplantation, primary biliary cholangitis, primary sclerosing cholangitis

## Abstract

Primary biliary cholangitis (PBC) and primary sclerosing cholangitis (PSC) are chronic cholestatic liver diseases characterized by bile duct injury that may progress to fibrosis, cirrhosis, liver failure, and malignancy and ultimately require liver transplantation. Although these diseases share several clinical features, they differ substantially in pathogenesis, clinical course, and therapeutic approaches. Ursodeoxycholic acid remains the standard first-line therapy for PBC; patients with an inadequate biochemical response may be treated with second-line agents such as seladelpar, elafibranor, or fibrates. In contrast, no approved therapy has been shown to slow disease progression in PSC. Emerging therapies primarily target symptom and complication management, and overall management focuses on surveillance and timely referral for liver transplantation. This review summarizes current approaches to risk assessment, treatment, symptom management, monitoring, and transplantation decision-making in both conditions.

**Main Points**

- Primary biliary cholangitis (PBC) and primary sclerosing cholangitis (PSC) are chronic cholestatic liver diseases characterized by progressive bile duct injury and an increased risk of liver failure but differ substantially in their pathogenesis, clinical course, and treatment strategies.
- Treatment of PBC has evolved beyond ursodeoxycholic acid to include newer second-line and symptom-directed therapies, whereas PSC continues to lack an approved disease-modifying therapy.
- Current management of both diseases depends not only on biochemical monitoring and risk stratification but also on surveillance, symptom control, and timely referral for liver transplantation when clinically indicated.
- Future studies in both diseases should incorporate patient-reported outcomes, as biochemical improvement does not always correlate with symptom relief or quality of life.

## Introduction

Primary biliary cholangitis (PBC) and primary sclerosing cholangitis (PSC) are rare chronic cholangiopathies characterized by immune-mediated injury of the bile ducts and progressive fibrosis. Over time, both conditions can lead to cirrhosis, hepatic decompensation, and hepatobiliary malignancy in the absence of effective management.^1^^,^^2^ PBC predominantly affects middle-aged women and is characterized by autoimmune injury of the small intrahepatic bile ducts, with typical association with antimitochondrial antibodies and lymphocytic cholangitis.^1^^,^^3^ PSC involves fibrosing inflammation of both intrahepatic and extrahepatic bile ducts and is frequently associated with inflammatory bowel disease.^2^^,^^4^ Despite sharing a cholestatic clinical profile, the natural history and therapeutic options of the 2 conditions differ substantially, requiring distinct management approaches based on current evidence.

In PBC, recent advances have enabled earlier diagnosis, improved risk stratification, and expanded treatment options. Ursodeoxycholic acid (UDCA) remains the standard first-line therapy and improves transplant-free survival; however, up to 40% of patients have an incomplete biochemical response.^5^^,^^6^ In such cases, additional therapy with second-line agents may be considered, often guided by prognostic tools such as the GLOBE and UK-PBC scores.^7^^,^^8^ PSC 0poses distinct therapeutic challenges, characterized by a highly variable disease course and the absence of any treatment proven to slow disease progression.^9^ Care is therefore focused on surveillance for cholangiocarcinoma (CCA) and colorectal cancer, management of complications, symptom control, and timely referral for transplantation when indicated.^10^ Several ongoing trials are evaluating mechanism-driven therapies, with early signals of improvement in biochemical markers and patient-reported symptoms.^11^^,^^12^ Recent studies have also placed increased emphasis on patient phenotypes and patient-reported outcomes when assessing treatment effects.^13^^-^^15^

The current review synthesizes current treatment goals, risk stratification, medical and supportive therapies, complication management, surveillance strategies, and transplant decision-making in PBC and PSC. Ongoing challenges and emerging therapeutic strategies are also discussed, particularly given the continued absence of disease-modifying options for PSC.

## Primary Biliary Cholangitis

### Epidemiology and Pathogenesis

PBC is a chronic autoimmune cholestatic liver disease with geographic variation. Global prevalence is approximately 18 cases per 100 000 persons, with an incidence of 1.8 per 100 000 persons per year.^16^ In the United States, prevalence reached 40.9 per 100, 000 adults in 2021.^17^ Current evidence suggests a multifactorial etiology involving genetic susceptibility and immune dysregulation.^18^ Several susceptibility loci have been identified,^19^ including human leukocyte antigen (HLA) class II genes such as HLA-DRA, interleukin-21 and its receptor (IL-21/IL-21R), the T-cell costimulatory pathway CD28/cytotoxic T-lymphocyte–associated protein 4 (CD28/CTLA4), and nuclear factor kappa B1 (NFκB1).^20^ Altered T-cell activity, dysregulated cytokine signaling,^21^ and injury to biliary epithelial cells—including disruption of the protective bicarbonate barrier—have also been described.^22^ This mechanistic understanding has shaped current therapeutic approaches.

### First-Line Management: Ursodeoxycholic Acid

UDCA consistently improves cholestatic biochemical parameters and long-term clinical outcomes and remains the first-line therapy for PBC after decades of investigation.^23^^,^^24^ The recommended dose is 13-15 mg/kg/day.^24^ UDCA improves cholestasis by increasing bile flow and reducing bile acid–mediated injury to hepatocytes and cholangiocytes.^24^ Early initiation of therapy has been associated with slower disease progression and improved transplant-free survival.^25^ Despite these benefits, 30%-40% of patients receiving UDCA monotherapy do not achieve an adequate biochemical response after 1 year of treatment.^26^^,^^27^ Persistent elevations in cholestatic markers reflect ongoing disease activity and are associated with an increased risk of disease progression, cirrhosis, and liver transplantation.^24^Recognition of this incomplete response has reshaped treatment strategies in PBC; patients with persistent biochemical activity despite optimal UDCA therapy are candidates for treatment escalation with second-line agents targeting additional pathways involved in cholestatic injury and disease progression.^7^^,^^28^

### Treatment Goals: Achieving Biochemical Response

The primary treatment goals in PBC are to achieve a biochemical response, slow disease progression, and prevent cirrhosis.^29^Biochemical response is defined by normalization or substantial reduction in cholestatic liver enzymes and bilirubin after 12 months of UDCA therapy. Earlier assessment may identify patients at higher risk who require treatment modification.^26^ Established biochemical response criteria help identify patients with an inadequate response to UDCA who may benefit from second-line therapy or enhanced monitoring.^27^^,^^30^ The POISE criteria define response as alkaline phosphatase (ALP) ≤1.67× ULN with at least a 15% reduction and normal bilirubin.^31^ The Paris I criteria define response as ALP <3× upper limit normal (ULN), aspartate aminotransferase (AST) <2× ULN, and normal bilirubin, whereas the Paris II criteria use stricter thresholds of ALP and AST <1.5× ULN and normal bilirubin.^30^^,^^32^ Other definitions include the Barcelona criteria (≥40% reduction or normalization of ALP), the Rotterdam criteria (incorporating albumin alongside ALP and bilirubin), and the Toronto criteria (ALP ≤1.67× ULN after 2 years of UDCA therapy).^25^^,^ ^30^^-^^32^

Beyond these criteria, baseline evaluation and stratification of high-risk patients are necessary to determine whether second-line treatment is warranted before the 12-month assessment point.^26^ High-risk patients are more often men younger than 55 years and are more likely to be symptomatic or to present with overlap or ductopenic variants.^33^ They typically exhibit worse baseline serologic, biochemical, histologic, and fibrosis-marker features, including elevated bilirubin, ALP ≥2× ULN, advanced fibrosis or cirrhosis, elevated or rising stiffness scores, positive anti-gp210 or anti-centromere antibodies, and features of autoimmune hepatitis (AIH).^34^^-^^35^

Normal ALP levels are associated with improved long-term outcomes, particularly in patients with advanced fibrosis or at a younger age.^36^ Deep biochemical response, an emerging concept in PBC, refers to near-normalization of key cholestatic markers, specifically ALP and bilirubin, following UDCA therapy.^37^ This level of biochemical control is associated with improved prognosis and is increasingly considered a clinically meaningful therapeutic target.^37^

Additional laboratory parameters contributing to risk assessment include low albumin, reduced platelet count, and elevated gamma-glutamyltransferase (GGT) or transaminases, which may reflect advanced disease or ongoing hepatocellular injury.^38^

### Estimating Transplant-Free Survival

Management of PBC also aims to prevent complications of advanced liver disease, reduce the risk of hepatocellular carcinoma (HCC) in high-risk patients, and address symptoms such as fatigue and pruritus.^38^^-^^40^ The GLOBE and UK-PBC scores are the most widely used instruments for estimating transplant-free survival in PBC.^41^^,^^42^ The GLOBE score incorporates age, bilirubin, albumin, ALP, and platelet count measured after 1 year of UDCA treatment and has demonstrated strong performance in both derivation and validation cohorts.^41^ The UK-PBC score estimates the risk of liver transplantation or liver-related death at 5, 10, and 15 years using baseline albumin and platelet count, along with bilirubin, transaminases, and ALP measured after 12 months of therapy.^42^Validation studies, including US-based cohorts, have reported strong predictive performance for both models.^7^^,^^43^ Additional tools, including the albumin-bilirubin score, the aspartate aminotransferase-to-platelet ratio index (APRI), the Mayo score, models developed for response to obeticholic acid (OCA), and the neutrophil-to-lymphocyte ratio, have also been evaluated for risk stratification in PBC.^44^^,^^45^

### Second-Line Therapies: Management of Ursodeoxycholic Acid Nonresponders

#### Fibrates:

Fibrates are peroxisome proliferator-activated receptor alpha (PPAR-α) agonists with anticholestatic and hepatoprotective properties.^46^ PPAR-α activation stimulates biliary phospholipid secretion (ABCB4/MDR3), reduces bile acid synthesis, inhibits NFκB–mediated inflammatory signaling, and modulates hepatic bile acid toxicity.^47^^-^^49^

In the phase III BEZURSO trial (n = 100), addition of bezafibrate 400 mg/day to UDCA resulted in a complete biochemical response in 31% of patients compared with none in the placebo group at 24 months (*P* < .001), with ALP normalization in 67% vs. 2%.^50^ Bezafibrate is approved for PBC in some European countries and Japan.^48^ It is generally well tolerated; however, myalgia occurred in 20% of patients compared with 10% in the placebo group, and creatinine increased by about 5% from baseline.^50^

Fenofibrate (160-200 mg/day) has demonstrated comparable biochemical efficacy in multiple studies.^51^ In a 2022 randomized trial (n = 48), fenofibrate 200 mg/day plus UDCA achieved ALP normalization in 54.2% of patients compared with 4.2% with UDCA alone at 12 months (*P* < .001), with complete biochemical response in 20.8% vs. 0%.^52^ Fenofibrate is frequently used off-label in settings where bezafibrate is unavailable, including in the United States.^48^

#### Obeticholic Acid:

OCA is a farnesoid X receptor (FXR) agonist that regulates bile acid synthesis, transport, and hepatic inflammation by suppressing CYP7A1 and promoting bile acid export from hepatocytes.^53^^,^^54^ It was approved in 2016 as second-line therapy for PBC based on the POISE trial. Treatment is initiated at 5 mg daily and may be increased to 10 mg once daily if tolerated and if ALP remains elevated.^53^^,^^54^ Clinical studies showed reductions in predicted 10-year event risk of 26% using GLOBE scores and 37% using UK-PBC scores, with ALP normalization in 7%-25% of patients during follow-up.^55^^,^^56^ Despite this efficacy, clinical use has been limited by tolerability and safety concerns. Pruritus occurred in 50%-70% of patients, and discontinuation rates reached 45% at 48 months.^53^^-^^55^ Reports of hepatotoxicity in patients with advanced cirrhosis prompted a request by the US Food and Drug Administration (FDA) for market withdrawal; OCA was no longer available in the United States as of November 14, 2025.^55^^,^^57^ The European Medicines Agency (EMA) revoked OCA’s marketing authorization in the European Union (EU) on June 28, 2024, after concluding that confirmatory data failed to demonstrate meaningful clinical benefit beyond biochemical improvement.^58^ Prescribing practices in the United Kingdom were not affected, as the UK regulatory authority operates independently of the EMA.^59^

#### Elafibranor:

Elafibranor is a dual PPAR-α/δ agonist that received FDA accelerated approval in 2024. It is administered orally once daily at 80 mg and targets metabolic and inflammatory pathways involved in cholestasis.^60^^-^^62^ In the phase III ELATIVE trial (n = 161) of patients with an inadequate response to UDCA, 51% of those receiving elafibranor achieved the primary composite biochemical endpoint at 52 weeks compared with 4% receiving placebo, with ALP normalization in 15% vs. 0%.^63^^,64^ ALP reductions exceeded 140 U/L compared with UDCA monotherapy, whereas bilirubin levels remained stable or improved.^63^ Modest improvements in pruritus were also observed, and exploratory long-term data suggested clinically meaningful reductions in fatigue, particularly among patients with moderate-to-severe symptoms at baseline.^65^^,^^66^ Elafibranor is generally well tolerated; the most commonly reported adverse effects are mild gastrointestinal symptoms such as nausea, diarrhea, and abdominal pain, and discontinuation rates are approximately 10%.^60^^-^^66^However, caution is warranted in women of childbearing potential, given evidence of fetal harm in animal studies.^67^

#### Seladelpar:

Seladelpar is a selective PPAR-delta (δ) agonist that received FDA accelerated approval in 2024.^68^ It is administered orally once daily at 10 mg and improves bile acid homeostasis, lipid metabolism, and inflammatory signaling.^69^^-^^72^ In the phase III RESPONSE trial (n = 297) of patients with an inadequate response to UDCA, 61.7% of those receiving seladelpar achieved the primary biochemical endpoint at 12 months compared with 20% receiving placebo, with ALP normalization in 25% vs. 0%.^69^^,^^72^^,^^73^ Furthermore, treatment reduced cholestatic pruritus, with improvements of 1.5-2.0 points on the pruritus numerical rating scale and a lower overall incidence of pruritus (relative risk: 0.54).^69^^,^^73^ Long-term data from the ASSURE extension study showed sustained biochemical response beyond 2 years, with ALP normalization in approximately 30% of patients and discontinuation rates of 5%-8%.^69^^,74^ Reported adverse events include headache, nausea, and diarrhea; the overall safety profile is similar to placebo, and no significant hepatotoxicity signals have emerged.^69^^,^^72^^-^^74^An ongoing trial (NCT06051617) is evaluating seladelpar in patients with PBC and compensated cirrhosis, representing a potential therapeutic option for a population with previously limited alternatives.^75^

### Symptom-Directed Therapy

#### Management of Pruritus:

Cholestatic pruritus affects approximately 70% of patients with PBC and is among the most challenging symptoms to treat.^76^ Bile acids, lysophosphatidic acid, and autotaxin are thought to contribute to pruritogenesis, although symptom severity does not reliably correlate with standard laboratory markers of cholestasis.^77^^,^^78^Initial therapy involves bile acid sequestrants, primarily cholestyramine (4-16 g daily in divided doses), which binds bile acids in the intestine and reduces their recirculation.^78^ Its clinical use is often limited by gastrointestinal adverse effects and palatability; it should be administered separately from other medications to avoid drug binding.^78^Rifampin (150-300 mg twice daily) may be used when symptoms persist, with monitoring of liver enzymes given the potential for transaminase elevation.^78^^,^ ^79^

Additional options include the opioid antagonist naltrexone (25-50 mg daily) and the selective serotonin reuptake inhibitor sertraline (75-100 mg daily), although evidence of benefit is variable. ^77^^-^^79^Ileal bile acid transporters (IBAT) inhibitors are under investigation as novel treatments. In the phase III GLISTEN trial, linerixibat demonstrated greater reductions in pruritus scores than placebo at 24 weeks, contributing to its recent US FDA approval on March 19, 2026, as the first therapy specifically approved for cholestatic pruritus in PBC.^77^^,^^78^^,80^ Maralixibat is also under investigation.^77^^,^^78^Volixibat is being evaluated in phase 2 studies that have demonstrated reductions in pruritus and bile acid concentrations.^80^ Seladelpar has also shown improvements in pruritus in recent clinical trials.^69^^,^^72^^,^^73^ For patients with severe, refractory symptoms, procedural interventions such as plasmapheresis or nasobiliary drainage may be considered.^79^

#### Management of Fatigue:

Fatigue affects more than half of patients with PBC and is among the most challenging symptoms to manage, as no medication has demonstrated consistent benefit.^81^^,^^39^Its pathophysiology is multifactorial and incompletely understood, with proposed mechanisms including autonomic dysfunction, sleep disturbance, and impaired skeletal and cardiac muscle bioenergetics.^39^ Elevation of inflammatory cytokines, progesterone metabolites, and molecular markers such as brain-derived neurotrophic factor are under investigation as contributing pathways.^82^Assessment is supported by validated instruments such as the Fatigue Severity Scale (FSS), which quantifies patients’ perceived fatigue; higher scores indicate greater fatigue severity, with a commonly used clinical threshold of >4 identifying significant fatigue.^83^ Higher FSS scores have been associated with worse clinical outcomes and greater symptom burden, supporting the utility of the scale as a reliable measure of fatigue severity in patients with PBC.^83^ Management relies on supportive measures, including sleep hygiene, gradual physical activity, and treatment of contributing comorbidities such as hypothyroidism, anemia, and depression.^39^ Golexanolone, a neurosteroid modulator targeting GABA-A receptor signaling, is being evaluated in an ongoing phase 1b/2 trial (NCT07304843) in patients with PBC-associated fatigue and cognitive dysfunction, representing a novel investigational approach to one of the most treatment-refractory symptoms in PBC.^84^

#### Management of Other Symptoms and Complications:

Sicca symptoms are common, with dry mouth and dry eyes affecting 30%-50% of patients, occasionally in association with Sjögren’s syndrome.^85^Xerostomia is managed with saliva substitutes, sugar-free gum, frequent water intake, and secretagogues such as pilocarpine or cevimeline.^85^Xerophthalmia is treated with lubricating drops or gels, and more severe cases may require punctal plugs or topical cyclosporine.^85^

Bone health requires ongoing attention in long-term management. Osteoporosis occurs in up to 40% of patients and is associated with vitamin D malabsorption and chronic inflammation.^85^^,^^86^ Bone mineral density testing is recommended at diagnosis and every 2-4 years thereafter. Treatment includes vitamin D and calcium supplementation, weight-bearing exercise, and bisphosphonate therapy for patients with established osteoporosis or increased fracture risk.^87^

Lipid abnormalities are frequently observed because of cholestasis and the accumulation of lipoprotein X.^88^ Despite this lipid profile, cardiovascular risk does not appear to be increased. Statins are considered safe in PBC and may be used when traditional cardiovascular risk factors are present.^85^

### Liver Transplantation

Liver transplantation is the definitive treatment for advanced PBC.^89^ It is indicated in patients with decompensated cirrhosis, MELD score ≥15 in MELD-based allocation systems, with regional variations in listing and allocation criteria, including Turkey, HCC within Milan criteria, or refractory pruritus unresponsive to medical therapy.^89^^,^^90^Ten-year survival after liver transplantation for PBC approaches 80%, although disease recurrence is reported in 17%-46% of patients and may affect graft and patient outcomes.^90^The use of UDCA after transplantation has been associated with reduced PBC recurrence and improved graft and patient survival.^91^ In PBC, calcineurin inhibitor–based therapy remains central after liver transplantation, but the optimal agent remains debated, with some studies suggesting higher recurrence risk with tacrolimus and potential protection with cyclosporine. However, recent registry data found no survival or recurrence-related graft loss benefit with cyclosporine over tacrolimus, supporting individualized immunosuppression.^92^

## Primary Sclerosing Cholangitis

### Epidemiology and Pathogenesis

PSC is a chronic cholangiopathy characterized by progressive inflammation and fibrosis of the intrahepatic and extrahepatic bile ducts, ultimately leading to cirrhosis and increased liver-related morbidity and mortality.^93^^,^^94^ PSC is a rare disease with geographic variation; population-based studies report an incidence of approximately 0.6-0.9 cases per 100 000 person-years and a prevalence of approximately 13 cases per 100 000 persons.^95^ The disease predominates in men, with a male-to-female ratio of approximately 2:1, and the mean age at diagnosis is approximately 40 years.^95^ Current evidence supports a multifactorial pathogenesis involving immune-mediated injury in genetically susceptible individuals.^95^^,^^14^

**Figure 1.**
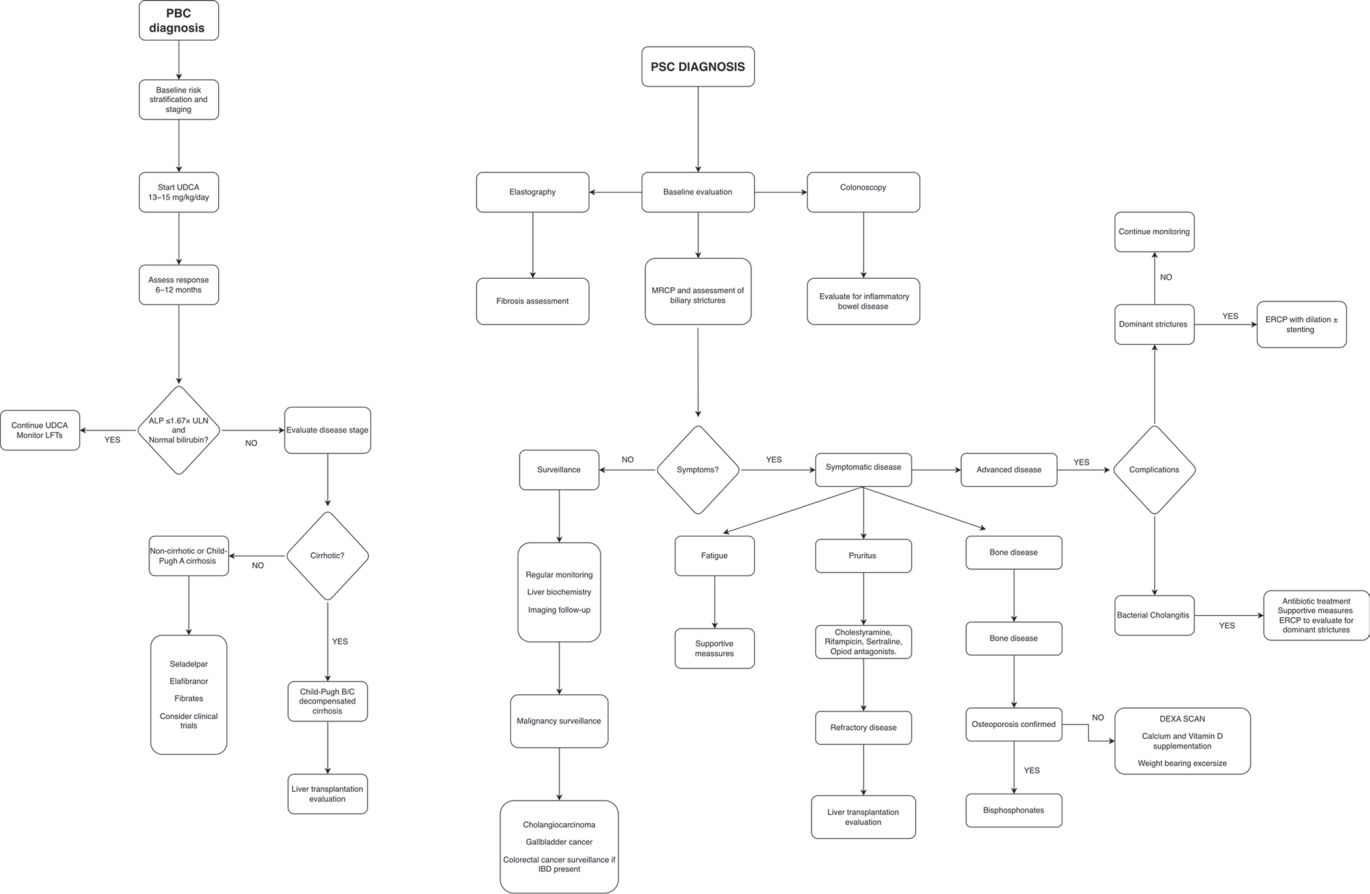
Management algorithms for A) primary biliary cholangitis (PBC) and B) primary sclerosing cholangitis (PSC). This figure summarizes the clinical management pathways for PBC and PSC based on current guideline recommendations from the European Association for the Study of the Liver and the American Association for the Study of Liver Diseases.^1^^-^^4^,^93^^,^^94^ Abbreviations: ALP, alkaline phosphatase; ERCP, endoscopic retrograde cholangiopancreatography; IBD, inflammatory bowel disease; MRCP, magnetic resonance cholangiopancreatography; PBC, primary biliary cholangitis; PSC, primary sclerosing cholangitis; UDCA, ursodeoxycholic acid.

Genetic studies have identified associations within the HLA region, particularly the HLA-DRB1*03* (*DR3*) and *HLA-DRB1*13 haplotypes, suggesting that variants at this locus contribute to disease susceptibility and disease progression.^14^ PSC is clinically heterogeneous and includes several phenotypes defined by bile duct involvement and associated conditions. Large-duct PSC is the most common form and is characterized by multifocal strictures and segmental dilatations of the intrahepatic and/or extrahepatic bile ducts on cholangiography. Small-duct PSC occurs in patients with biochemical and histologic features of PSC but normal cholangiography findings and is generally associated with a more favorable prognosis.^94^^-^^96^^,^^14^ Additional recognized phenotypes include PSC associated with inflammatory bowel disease, immunoglobulin G4-related PSC, and PSC complicated by relevant strictures (RS), each of which may influence the clinical course and management (Figure 1).^94^^,^^95^^,^^14^^,^^96^

### Pharmacologic Treatment: Current Evidence

Several prognostic models have been developed for PSC, including the Amsterdam-Oxford model, UK-PSC score, Mayo risk score, and Primary Sclerosing Cholangitis Risk Estimate Tool (PrEsTo). Unlike in PBC, where validated models such as GLOBE and UK-PBC are widely used to estimate long-term risk and treatment response, prognostication in PSC remains more limited because of disease heterogeneity, variable endpoints, fluctuating cholestatic markers, and the unpredictable risk of complications such as cholangitis and cholangiocarcinoma. Although newer tools such as UK-PSC and PREsTo may improve risk stratification compared with the Mayo risk score, their use remains primarily supportive and should not replace individualized clinical assessment.^97^^,^^98^

#### Ursodeoxycholic Acid in Primary Sclerosing Cholangitis:

Several pharmacologic therapies have been investigated for PSC, including UDCA, antibiotics, immunosuppressive agents, and, more recently, treatments targeting the gut microbiome.^96^ UDCA has been the most extensively studied and has been used clinically since the early 1990s, when randomized trials demonstrated improvements in ALP and aminotransferase levels with doses of 13-15 mg/kg/day.^99^ However, higher doses (28-30 mg/kg/day) are contraindicated because a randomized controlled trial identified serious adverse effects and did not support their empiric use in patients with PSC.^100^

#### Emerging Therapies:

Recent clinical trials have shifted toward mechanism-driven designs, with several studies actively enrolling participants since 2022. These trials focus on validated surrogate endpoints, patient-reported outcomes, and long-term disease modification.^101^

#### Norursodeoxycholic Acid:

Among bile acid-based strategies, norursodeoxycholic acid (norUDCA; 500-1500 mg/day) remains under evaluation following encouraging phase II data and is currently being investigated in a large international phase III trial NUC-5 (NCT03872921).^102^ Early studies demonstrated improvements in ALP levels and the potential attenuation of histologic disease progression, with benefits observed across multiple secondary outcomes and a safety profile comparable to that of placebo.^102^

NorUDCA is a modified bile acid derived from UDCA with anti-inflammatory and antifibrotic properties that promote cholehepatic shunting and improve bile flow.^103^ Randomized clinical trials have demonstrated dose-dependent biochemical improvements; however, an effect on disease progression has not yet been established. The compound remains investigational.^103^

#### Immunosuppressive and Anti-inflammatory Therapies:

Immunosuppressive and anti-inflammatory therapies have also been investigated for PSC. Agents such as prednisone, prednisolone, azathioprine, methotrexate, and mycophenolate mofetil have been evaluated in small clinical trials and observational studies.^104^These studies have not demonstrated consistent clinical benefit in patients with isolated PSC, therefore, these agents are not recommended as part of routine clinical management. An exception is the PSC–AIH overlap syndrome, for which management follows established AIH guidelines.^104^

#### Gut–Liver Axis in Primary Sclerosing Cholangitis:

The role of the gut–liver axis in PSC pathogenesis has prompted the evaluation of microbiome-targeted therapies.^105^ Oral antibiotics, including vancomycin and metronidazole, have been studied in small randomized and open-label trials and have demonstrated reductions in ALP levels. Subsequent studies have not confirmed benefits with respect to disease progression or long-term clinical outcomes; therefore their use remains limited to clinical trial settings.^105^ Fecal microbiota transplantation has also been investigated as a microbiome-modifying strategy in PSC, with early pilot studies suggesting safety and increased microbial diversity. However, its therapeutic efficacy remains preliminary and unproven.^106^

#### Peroxisome Proliferator–Activated Receptors in Primary Sclerosing Cholangitis:

Elafibranor has been investigated as a therapeutic candidate for PSC because of its effects on bile acid metabolism, inflammation, and hepatic fibrosis.^107^ In a phase II randomized trial (NCT05627362), elafibranor treatment resulted in dose-dependent reductions in ALP levels and improvements in other cholestatic markers at 12 weeks, with the higher dose also associated with improvement in pruritus. Treatment was generally well tolerated, and most adverse events were mild. Bezafibrate, another PPAR agonist, has also been evaluated in PSC. Early studies demonstrated reductions in hepatobiliary enzymes and improvements in liver biochemistry in a subset of patients; however, evidence remains limited and its impact on long-term disease progression has not been established. Whether these biochemical improvements translate into modification of long-term disease progression requires confirmation in larger, longer-duration studies.^108^

#### Nonsteroidal Farnesoid X Receptor Agonists:

Nonsteroidal FXR agonists, including cilofexor and tropifexor, have been evaluated in phase II trials conducted between 2022 and 2024 and have demonstrated reductions in cholestatic biomarkers. Prognostic benefit remains unestablished. Safety concerns and tolerability issues have limited their broader clinical application.^109^

#### Ileal Bile Acid Transporter Inhibitors:

IBAT inhibitors act in the terminal ileum to block bile acid reabsorption and interrupt the enterohepatic circulation. This mechanism reduces circulating bile acid concentrations and may help relieve cholestasis and pruritus. Several agents, including linerixibat, maralixibat, and volixibat, are being investigated in cholestatic liver diseases such as PSC. Early clinical studies have reported improvements in pruritus and reductions in bile acid concentrations; however, diarrhea and other gastrointestinal effects have been frequently observed.^110^

#### Antifibrotic Therapies:

CM-101, a monoclonal antibody targeting CCL24, is currently being evaluated in a phase II randomized trial, with primary endpoints including ALP levels, fibrosis markers, and patient-reported outcomes. This agent represents an emerging antifibrotic strategy for PSC (NCT04595825).^111^

### Management of Complications

The most frequent complications are RS, recurrent bacterial cholangitis, and hepatobiliary malignancies, particularly CCA.^93^^,94^ RS are defined as narrowing of the common bile duct to ≤1.5 mm or of a hepatic duct to ≤1.0 mm on cholangiography. They contribute significantly to symptom burden and are associated with adverse outcomes and rapid clinical deterioration. Clinical context and symptoms remain essential in determining their significance.^112^

Recurrent bacterial cholangitis presents with worsening cholestasis, jaundice, or systemic inflammatory symptoms. These episodes should prompt evaluation and management, including antibiotic therapy and clinical assessment for possible endoscopic intervention in the setting of RS.^93^^,^^94^^,^^96^^,^^112^ Endoscopic retrograde cholangiopancreatography (ERCP) is reserved for complication management rather than disease modification in selected patients with PSC.^113^^,^^114^ The procedure provides biliary decompression and enables tissue sampling, including brush cytology and, when available, fluorescence in situ hybridization. Balloon dilation is the preferred treatment for RS, and antibiotic prophylaxis is recommended for all patients undergoing ERCP because of the risk of infectious complications.^113^^,^^114^ However, the sensitivity of current techniques for the detection of malignancy remains limited.^113^^,^^114^

### Surveillance Strategies

The risk of malignancy is substantially increased in patients with PSC and represents a major determinant of morbidity and mortality, even in early-stage disease. PSC is associated with a markedly increased risk of CCA (estimated at 5%-15%), with many cases diagnosed within the first few years after diagnosis. Early detection remains challenging because CCA lacks a standardized diagnostic algorithm and can be difficult to distinguish from benign RS on imaging.^93^^,^^94^^,^^96^

Current guidelines support annual hepatobiliary surveillance using magnetic resonance imaging and magnetic resonance cholangiopancreatography, often in combination with CA 19-9, in adults, while acknowledging the limited sensitivity and specificity of this approach.^115^ ERCP is not recommended for routine surveillance and should be reserved for patients with clear clinical indications, including progressive cholestasis, jaundice, recurrent bacterial cholangitis, or suspected CCA.^115^

The risk for colorectal cancer is also increased, particularly among the 50%-80% of patients with concomitant inflammatory bowel disease. This increased risk supports the initiation of intensified colonoscopic surveillance at the time of PSC diagnosis, regardless of disease stage.^93^^,^^94^^,^^96^

### Estimating Transplant-Free Survival

#### Liver Transplantation in Primary Sclerosing Cholangitis:

Liver transplantation is the only intervention capable of replacing the irreversibly damaged liver in PSC. Approximately 40%-50% of patients develop liver-related complications or are listed for transplantation during the course of the disease.^93^^,^^94^^,^^96^ In addition to standard indications such as decompensated cirrhosis, declining liver function, and MELD-based allocation, liver transplantation may also be considered in PSC for recurrent bacterial cholangitis refractory to medical management, severe pruritus unresponsive to therapy, progressive cholestasis that significantly affects quality of life, and selected cases of early-stage CCA. The MELD score does not reliably reflect disease burden in PSC because patients may experience substantial symptoms and complications despite relatively preserved liver synthetic function.^116^ Post-transplant outcomes are generally favorable, with reported 5-year survival rates of 80%-85% and significant improvements in quality of life. Recurrence of PSC in the liver allograft occurs in roughly 20%-30% of cases and usually progresses slowly, with limited effects on medium-term graft survival.^116^

Risk factors for recurrent PSC after liver transplantation include underlying IBD, particularly active ulcerative colitis. The role of colectomy in modifying recurrence risk remains controversial. Earlier observational studies suggested that colectomy before or at the time of transplantation was less common among patients who developed recurrent PSC, raising the possibility of a protective association.^117^ However, in the large European Liver Transplant Registry cohort, Visseren et al^118^ reported that recurrent PSC occurred in 16.7% of transplant recipients and was associated with worse graft and patient survival. Registry-level data did not establish the timing of colectomy as a definitive protective strategy. Therefore, given the retrospective design of available studies, heterogeneity in patient populations and colectomy timing, and lack of prospective evidence, colectomy cannot currently be recommended solely to reduce the risk of recurrent PSC.

Post-transplant immunosuppression does not appear to reliably prevent PSC recurrence because recurrent disease may develop despite systemic immunosuppression after liver transplantation. Therefore, immunosuppressive therapy should be guided by standard post-transplant, IBD, or overlap-syndrome indications rather than being used specifically to prevent PSC progression or recurrence.^119^

## Future Directions in Primary Biliary Cholangitis and Primary Sclerosing Cholangitis

In PBC, therapeutic development has expanded well beyond traditional bile acid–based approaches. Current and investigational therapies are summarized in Table 1 and include UDCA and UDCA-based combinations such as UDCA plus fenofibrate; PPAR-targeted agents such as elafibranor, saroglitazar, and fenofibrate; FGF19 pathway agents such as NGM282; IBAT inhibitors such as linerixibat; and immunomodulatory, anti-inflammatory, or antifibrotic agents including CNP-104, setanaxib, and rituximab. Although several agents have demonstrated improvements in ALP levels, pruritus, aminotransferase levels, autoantibody titers, or fibrosis-related biomarkers, many remain investigational and require larger, longer-term studies to determine whether biochemical responses translate into durable clinical benefit.^120^^,^ ^121^

Therapeutic research in PSC targets multiple pathogenic pathways, including bile acid metabolism, cholestatic pruritus, immune-mediated inflammation, hepatic fibrosis, and the gut–liver axis. Therapies under investigation are summarized in Table 1 and include UDCA and UDCA-based combination therapies such as UDCA plus fenofibrate; bile acid and nuclear receptor–targeted therapies including norUDCA, OCA, cilofexor, and NGM282/M70; PPAR agonists such as elafibranor and fenofibrate; IBAT inhibitors including volixibat and maralixibat; antibiotics such as vancomycin; and immunomodulatory, anti-inflammatory, or antifibrotic agents including vidofludimus calcium, bexotegrast, and CM-101. Although several agents have demonstrated improvements in ALP levels, pruritus, bile acid synthesis, or fibrosis-related biomarkers, most remain investigational and require larger, longer-term studies to determine whether biochemical responses translate into meaningful clinical benefit.For a summary of approved and investigational pharmacologic therapies in PBC and PSC with references, see Table 1.

Future clinical trials in cholestatic liver diseases should also systematically incorporate patient-reported outcomes (PROs) as primary study endpoints. Many previous studies have focused predominantly on biochemical markers; however, these measures do not fully capture patients’ symptom burden or functional status. Patients with both PBC and PSC continue to experience reduced health-related quality of life even after achieving biochemical normalization.^122^

The integration of validated PRO instruments alongside traditional laboratory and imaging endpoints will be essential for the comprehensive evaluation of treatment efficacy and the advancement of patient-centered therapeutic development, particularly given the persistent dissociation between biochemical and symptomatic outcomes observed in both diseases.^120^^-^^123^

## Conclusion

Treatment options for PBC and PSC have expanded in recent years, but important gaps remain. Biochemical improvements do not always translate into better symptoms or quality of life, and reliable surrogate markers for disease progression remain limited. Future clinical trials should incorporate patient-reported outcomes alongside biochemical and clinical endpoints. The development of effective disease-modifying therapies is still needed to reduce reliance on liver transplantation, which continues to be the only definitive treatment for end-stage disease.

PBC now has a broader, response-based treatment approach with options for escalation, whereas PSC still lacks effective disease-modifying therapies. These differences highlight the need for continued translational and clinical research, especially in PSC, to improve outcomes and address unmet needs.

## Figures and Tables

**Table 1.** Future Directions of Therapeutic Targets and Pharmacologic Therapies in Primary Biliary Cholangitis and Primary Sclerosing Cholangitis

**PBC/PSC**	**Therapy**	**Mechanism of Action**	**Stage **	**Dosage**	**Primary Endpoints**	**Adverse Effects**
PBC	UDCA^121^	Hydrophilic bile acid that increases bile flow and reduces bile acid–mediated hepatocyte injury	First-line approved therapy	13-15 mg/kg/day	↓ ALP, ↓ bilirubin	Generally well tolerated; mild weight gain, diarrhea
	Seladelpar^121^^,^^124^	Selective PPAR-δ agonist modulating bile acid metabolism and inflammation	Second-line approved therapy /Phase 3 ASSURE trial, NCT03301506	5-10 mg/day	↓ ALP, normalization of bilirubin, ↓ pruritus scores	Headache, abdominal pain, mild transaminase elevations
	Elafibranor^121^^,^^125^	Dual PPAR-α/δ agonist affecting bile acid metabolism and inflammatory signaling	Second-line approved therapy Phase 3 ELATIVE trial, NCT04526665	80 mg/day	↓ ALP, biochemical response	GI symptoms, transient transaminase elevation
	UDCA* + *Bezafibrate^121^^,^^126^	PPAR-α agonists improving bile acid metabolism	Phase 3 Bezurso trial, NCT01654731	400 mg/day	↓ ALP, ↓ GGT	Myalgia, renal dysfunction, increased creatinine
	UDCA* +*Fenofibrate^121^^,^^127^	PPAR-α agonists improving bile acid metabolism	Phase 3 NCT02823353	200 mg/day	↓ ALP, ↓ GGT	Myalgia, renal dysfunction, increased creatinine
	Obeticholic acid^57^^,^^121^^,^ ^128^	Farnesoid X receptor (FXR) agonist reducing bile acid synthesis	Former second-line therapy Phase 3 POISE trial, NCT01473524	5-10 mg/day	↓ ALP, ↓ bilirubin	Postmarketing safety concerns led to regulatory restrictions and withdrawal for PBC in the United States
	Saroglitazar^121^^,^^129^	PPAR-α/PPAR-γ	Phase 2/Phase 3 NCT05133336	1-2 mg/day	No summary efficacy results or structured adverse-event data have been published.	
	CNP-104^130^	Nanoparticle immunotherapy	Phase 2 NCT05104853	4 mg/kg or 8 mg/kg IV infusion (Day 1/Day 8)	No summary efficacy results or structured adverse-event data have been published.	
	NGM282^131^	FGF19 analog	Phase 2 NCT02026401	0.3-3 SC mg/day	↓ ALP, ↓ transaminases	Injection-site reactions, GI symptoms
	Setanaxib^132^	NOX1/NOX4 inhibitor	Phase 2/3 NCT03226067	400/1600 mg/day	↓ ALP, ↓ fibrosis	Headache, fatigue, GI symptoms
	Rituximab^133^	Anti-CD20	Phase 2 NCT00364819	IV infusion	↓ Autoantibodies	Infusion reactions, infections, fatigue, headache
	Linerixiba ^80^^,^^134^	IBAT inhibitor	Phase 3 GLISTEN NCT04950127	80 mg/day	↓Pruritus	GI symptoms
PSC	UDCA^12^^,^^99^^,^^101^	Hydrophilic bile acid improving bile flow	Randomized clinical trials	13-15 mg/kg/day	↓ ALP	No clinical benefit; high-dose therapy associated with serious adverse events
	UDCA + Fenofibrate^12^	Hydrophilic bile acid improving bile flow + PPAR agonist	Phase 3 IRCT2020042704722	UDCA (13-15 mg/kg/day) + 200 mg/day	No summary efficacy results or structured adverse-event data have been published.	
	NorUDCA^135^^,^^136^	Modified bile acid promoting cholehepatic shunting and bile flow	Phase 2/Phase 3 NCT01755507 NCT03872921	500-1500 mg/day	↓ ALP	GI symptoms
	Obeticholic acid^137^	FXR agonist regulating bile acid synthesis	Phase 2 NCT02177136	(1.5-3.0 mg/day or 5-10mg/day)	↓ ALP	Dose-related pruritus, GI symptoms, fatigue, pyrexia, fatigue
	Elafibranor^12^^,^^138^	Dual PPAR-α/δ agonist affecting bile acid metabolism and inflammatory signaling	Phase 2 ELMWOOD NCT05627362	80-120 mg/day	↓ ALP, ↓ fibrosis	GI symptoms, fatigue, headache
	Cilofexor^139^	Nonsteroidal FXR agonist regulating bile acid synthesis	Phase 2 NCT02943460	30-100 mg/day	↓ ALP, ↓ GGT, ↓ Fibrosis	Pruritus, fatigue
	NGM282 (M70)^140^	FGF19 analogs	Phase 2 NCT02704364	1-3 mg/day	↓ Fibrosis ↓ Bile acid synthesis	GI symptoms, hepatobiliary disorders, infections, increased billirubin
	Vidofludimus calcium^141^	Immunosuppressant	Phase 2 NCT03722576	15-30 mg/day	ALP normalization	GI symptoms, fatigue, pyrexia, pruritus
	Volixibat^142^	IBAT inhibitor	Phase 2 NCT04663308	40-160 mg/day	No summary efficacy results or structured adverse-event data have been published	
	Maralixibat^143^	IBAT inhibitor	Phase 2 NCT02061540	0.5-10 mg/day	↓ Pruritus	GI symptoms
	Vancomycin^144, 145^	Antibiotic	Phase 2/3 NCT03710122 NCT01802073	1.5-3 g/day	No summary efficacy results or structured adverse-event data have been published.	
	Bexotegrast^146^	αvβ6/αvβ1 integrin inhibitor	Phase 2 NCT04480840	40-320 mg/day	↓ ALP, ↓ fibrosis	GI symptoms, fatigue, upper respiratory tract infections, headache
	CM-101^147^	Monoclonal antibody targeting CCL24 to reduce inflammation and fibrosis	Phase 2 NCT04595825	10-20 mg/kg IV every 3 weeks	↓ ALP, ↓ fibrosis	Infusion reactions, mild systemic symptoms

This table summarizes approved and investigational pharmacologic therapies targeting key pathogenic pathways in PBC and PSC, including thier mechanisms of action, stages of development, dosing regimens used in clinical trials, main biochemical endpoints, and reported adverse effects. Unless otherwise specified, all doses refer to oral administration. Data are derived from published clinical trials and recent review articles.

ALP, alkaline phosphatase; CCL24, C-C motif chemokine ligand 24; CD20, cluster of differentiation 20; FGF19, fibroblast growth factor 19; FXR, farnesoid X receptor; GGT, gamma-glutamyl transferase; GI, gastrointestinal; IBAT, ileal bile acid transporter; IV, intravenous; NCT, National Clinical Trial identifier; NOX, nicotinamide adenine dinucleotide phosphate oxidase; norUDCA, norursodeoxycholic acid; OCA, obeticholic acid; PBC, primary biliary cholangitis; PPAR, peroxisome proliferator-activated receptor; PSC, primary sclerosing cholangitis; SC, subcutaneous; UDCA, ursodeoxycholic acid.

## Data Availability

The data that support the findings of this study are available on request from the corresponding author.
